# From separate streams to confluence: A framework for meaningful mixed methods integration in African primary care research

**DOI:** 10.4102/phcfm.v17i2.5201

**Published:** 2025-11-24

**Authors:** Robin E. Dyers, Kéfilath Bello, Timothy C. Guetterman

**Affiliations:** 1 Division of Health Systems and Public Health, Department of Global Health, Faculty of Medicine and Health Sciences, Stellenbosch University, Cape Town, South Africa; 2 WHO-FIC Collaborating Centre, Burden of Disease Research Unit, South African Medical Research Council, Cape Town, South Africa; 3 Department of Health and Wellness, Western Cape Government, Cape Town, South Africa; 4 Centre for Research in Human Reproduction and Demography, Cotonou, Benin; 5 Department of Family Medicine, University of Michigan, Ann Arbor, United States of America

**Keywords:** mixed methods, African health research, primary health care, integration framework, implementation research, health systems research

## Abstract

Mixed methods research is becoming more common in African primary care studies, yet systematic reviews show most mixed methods studies demonstrate low methodological rigour in integrating qualitative and quantitative components. This integration failure undermines addressing complex health challenges facing African primary care systems, where medical pluralism, resource constraints, and diverse disease burdens demand sophisticated methodological synthesis. This article describes a framework for achieving meaningful mixed methods integration in African primary care research contexts, addressing key challenges and providing practical strategies for transformative synthesis. Drawing on recent methodological advances, including the Mixed Methods Integration Quality Framework, systematic reviews of African mixed methods studies, and exemplar cases from primary care research, the framework integrates theoretical foundations with practical applications in resource-constrained settings. The framework encompasses: temporal considerations for integration decisions; identification of interface points; practical strategies including joint displays and data transformation; team-based approaches to synthesis; and solutions to common integration pitfalls. It addresses epistemological tensions, institutional barriers, and resource constraints in African research contexts. The framework enables researchers to move from separate methodological streams to genuine confluence, generating transformative insights transcending individual methodological contributions. By addressing context-specific challenges while maintaining methodological rigour, it supports producing nuanced understanding necessary for strengthening African health systems. This framework addresses integration challenges in African primary care research, offering practical tools for doctoral researchers and established investigators navigating complex health phenomena in resource-constrained settings.

## Introduction

While mixed methods research is common in health services, integrating findings from various approaches remains challenging. While researchers routinely combine quantitative and qualitative approaches, genuine synthesis that produces what Fetters and Freshwater term the ‘1+1=3’ effect continues to elude many studies.^1^ This integration challenge manifests particularly in African primary care research contexts, where systematic reviews have found that the majority of mixed methods studies demonstrate low methodological rigour in their integration of qualitative and quantitative components.^2^ The persistent gap between integration rhetoric and reality undermines the potential for mixed methods approaches to address the complex, multilayered health challenges facing African primary care systems.

Integration failure in mixed methods research represents more than methodological inadequacy; it constitutes a missed opportunity for generating a nuanced understanding necessary for effective health interventions. Recent evidence from sub-Saharan Africa reveals concerning patterns in mixed methods application, with systematic reviews finding that many studies fail to achieve integration at design, methods or interpretation levels despite the growing adoption of the mixed methods approach.^3^ The challenge intensifies for doctoral researchers who must navigate epistemological tensions between paradigms while developing technical competencies across traditions. Guetterman, Fetters and Creswell demonstrate that effective integration requires structured approaches such as joint displays to synthesise findings, yet emerging researchers face barriers including insufficient training in these techniques, limited supervisory expertise and institutional pressures favouring single-method outputs.^4^ These challenges compound in African academic contexts where resource constraints and limited methodological training opportunities create additional barriers to achieving sophisticated integration.

Primary care settings in Africa present unique considerations that both necessitate and complicate mixed methods integration. Health systems operate within complex adaptive environments characterised by medical pluralism, where biomedical services coexist with traditional healing systems, creating multiple therapeutic landscapes requiring diverse methodological lenses.^5^ Resource constraints paradoxically drive innovation, forcing researchers to develop efficient integrated data collection strategies that maximise insights while minimising burden on overstretched health facilities. Cultural diversity across and within African contexts demands flexible integration frameworks capable of accommodating different knowledge systems and values regarding health, illness and care. Furthermore, the complexity of health challenges in African settings requires methodological approaches sophisticated enough to capture interactions between disease patterns, health system responses and community dynamics.^5^

This article provides a comprehensive framework for achieving meaningful mixed methods integration specifically tailored to African primary care research contexts. Drawing on recent methodological advances, including the Mixed Methods Integration Quality Framework,^6^ we offer practical guidance addressing the unique challenges facing researchers and their supervisors. Our framework moves beyond abstract principles to provide concrete tools, strategies and exemplars demonstrating successful integration in resource-constrained settings, ultimately supporting the generation of transformative understanding necessary for strengthening African health systems.

## Understanding integration: Beyond the rhetoric

Integration encompasses purposeful synthesis, generating transformative insights that transcend individual methodological contributions.^6^ This requires interdependence that is connected, transactional, transformative, coherent and dynamic.^7^ Guetterman and Manojlovich term this ‘designing for integration’: methodological decisions throughout research intentionally creating synergistic opportunities.^8^

### Defining meaningful versus superficial integration

The Mixed Methods Integration Quality Framework distinguishes between presence and quality of integration.^6^ Meaningful integration requires systematic connection across study design, methodological procedures and interpretation/reporting levels. This contrasts with superficial mixing, where methods remain parallel, producing what Zhou and Wu identify as challenges in achieving meaningful integration, with researchers experiencing uncertainty during integration because of a lack of guidance and confidence.^9^

Recent evidence reveals concerning patterns. Systematic reviews find fewer than 20% of studies achieve meaningful integration.^9^ This pattern, noted earlier, particularly affects African contexts where basic triangulation often substitutes for genuine integration.^10^ The gap particularly affects resource-constrained settings where methodological training remains limited.^3^

### The integration continuum: Implications for practice

Contemporary frameworks conceptualise integration along continuums reflecting methodological interdependence.^7^ Minimal integration maintains independent methods with interpretation-stage comparison. Intermediate levels involve strategic connection points through sampling, instrument development or analytical direction.^4^ Meaningful integration achieves complete interdependence, generating emergent insights throughout.

This continuum holds particular relevance for resource-constrained contexts. African researchers demonstrate how constraints drive innovation.^11^ Recent studies employ ‘complexity-aware integration’: calibrating intensity to local conditions while maintaining rigour. This challenges assumptions that full integration always represents ideals.^6^ Researchers should assess their continuum position honestly, matching ambitions to available skills and resources.

### Misconceptions undermining integration quality

Contemporary literature identifies four persistent misconceptions.^9^ Temporal misconceptions position integration as analytical endpoints rather than continuous dialogue beginning with question formulation. Hierarchical misconceptions privilege quantitative validation over equitable paradigmatic dialogue.^6^ These manifest particularly in biomedically dominated research where qualitative insights are relegated to supporting roles.

Technical misconceptions conflate procedural techniques with interpretive synthesis. While joint displays and visual tools can serve as powerful analytical mechanisms for achieving integration through iterative development and meta-inference generation,^12,13^ their mere presence without analytical engagement does not guarantee meaningful integration.^9^ Resource misconceptions assume quality correlates with infrastructure, yet methodological rigour determines success.^8^ African researchers challenge these assumptions through community participation as integration mechanisms, demonstrating creativity matters more than material resources.^5^

### Structural barriers to integration achievement

Integration failure emerges from interconnected barriers documented in systematic reviews.^9,10^ Competence gaps leave researchers lacking cross-paradigmatic fluency. Training deficits compound challenges; only 15% of African universities provide dedicated integration training.^3^

Epistemological tensions create reconciliation challenges across knowledge production assumptions. Despite pragmatism’s promise, practical application remains challenging across disciplinary boundaries.^7^ Tensions intensify where biomedical and social science paradigms engage indigenous knowledge systems.^5^

Institutional structures systematically disadvantage integration. Publication constraints limit reporting space. Review processes lack mixed methods expertise.^9^ Funding undervalues synthesis. Academic evaluation privileges quantity over quality.^8^ These barriers interact with team dynamics where disciplinary hierarchies marginalise qualitative insights, particularly in biomedically dominated African primary care research.^11^

## Integration design decisions

Integration success depends on deliberate decisions about when and where to integrate methodologies throughout the research process.^4^ These decisions, made during study planning, determine whether integration produces transformative understanding or merely parallel findings.

### When to integrate: Temporal considerations

Design-phase integration establishes the foundation for meaningful synthesis. This involves crafting mixed methods research questions requiring both methodologies for complete answers, rather than questions addressable through single methods. African primary care researchers increasingly develop integrated conceptual frameworks bridging biomedical and socio-cultural perspectives from project inception, as Bekele et al. demonstrated in Ethiopian primary care.^5^ Critical decisions include determining methodological priority and sequence. Equal priority designs treat both methods as primary contributors, while unequal priority positions one method as dominant. In African contexts, these decisions often reflect institutional capabilities and interested parties’ expectations.

Sequential designs allow one method to inform another, particularly valuable when exploring unfamiliar phenomena. Convergent designs focus on bringing data or results together, often collecting data in similar time frames, which maximises efficiency in time-constrained settings but requires careful planning to ensure genuine integration rather than parallel implementation.^6^

Collection-phase integration creates opportunities for methodological dialogue while gathering evidence.^8^ African researchers demonstrate innovative approaches through community engagement where quantitative survey administration becomes an opportunity for qualitative observation, and focus groups inform survey refinement in real-time. Practical strategies include integrated sampling, where quantitative results identify qualitative participants. Data collection instruments designed for integration feature survey questions generating numerical data, while open-ended probes capture explanatory narratives.

Analysis-phase integration in African primary care research particularly values community validation sessions where interested parties interpret converging findings, ensuring analytical interpretations resonate with local realities and knowledge systems.^5,11^ This approach exemplifies how analysis-phase integration transforms separate data streams into a unified understanding through iterative cycles where findings from one method redirect analysis in another.^7^ Additional techniques include data transformation, cross-case analysis comparing patterns across methods, and following threads where surprising findings prompt targeted investigation. Team-based analysis, where researchers from different traditions jointly examine data, further strengthens the analytical process, demonstrating that sophisticated integration emerges from systematic analytical thinking.

Interpretation-phase integration synthesises findings into meta-inferences transcending individual methods.^9^ This involves identifying convergence, divergence and complementarity across findings, then theorising about patterns. Effective interpretation requires moving beyond listing separate findings to explaining relationships between them. African contexts add complexity as researchers must interpret findings across multiple knowledge systems, reconciling biomedical evidence with indigenous health concepts.

### Where to integrate: Points of interface

Mixed methods research questions serve as primary integration points. Effective questions explicitly identify how methods work together, using formulations like ‘how and to what extent’ or ‘what factors explain relationships’.^6^ African primary care research benefits from questions acknowledging complexity, examining both prevalence and meaning of traditional medicine use, or investigating how system metrics relate to patient experiences.

Integrated sampling strategies create natural connection points. Nested sampling embeds qualitative samples within quantitative populations. Parallel sampling selects comparable samples. Sequential sampling uses findings from one method to identify participants for another. African researchers navigate challenges including mobile populations, multilingual contexts and varying literacy levels.

Transformation points occur where data changes form to enable comparison.^4^ Quantitising converts qualitative data into numerical form through counting codes. Qualitising transforms quantitative data into narratives through profile development. These transformations require careful attention to maintaining data integrity.

Synthesis brings findings together through joint displays, integrated narratives or meta-inferences.^7^ Joint displays visually present findings side-by-side, revealing hidden patterns. Integrated narratives weave findings thematically. Meta-inferences articulate a new understanding emerging from integration. African primary care researchers increasingly employ participatory synthesis where communities contribute to interpretation, ensuring findings resonate with local realities while maintaining scientific rigour. These decisions about when and where to integrate ultimately determine whether mixed methods research achieves its transformative potential or remains merely additive, a distinction particularly salient in low-resource environments where every methodological decision carries opportunity costs.

Having established the critical decision points for integration, we now examine practical strategies for implementing these decisions in African primary care research contexts.

## Practical integration strategies

Joint displays have evolved substantially beyond simple side-by-side tables to become sophisticated analytical tools that actively facilitate integration rather than merely presenting it.^4^ The most effective displays emerge organically from the integration process itself, with their structure reflecting unique relationships discovered between findings rather than imposing predetermined formats.^1^ Joint displays that align statistics with themes are common but need careful design. Rather than mechanically placing numbers next to themes, researchers must explicitly articulate how statistical patterns relate to qualitative findings. Researchers explore whether and how themes connect to statistical patterns, using joint displays as analytical tools to discover relationships rather than assuming connections exist. This exploratory approach allows unexpected patterns to emerge while maintaining analytical rigour. Visual elements, including charts, maps and photographs, increasingly enrich these displays, proving particularly valuable for communicating integrated findings to diverse African health system interests.^11^

The iterative construction process itself generates integration insights as researchers discover connections, contradictions and gaps while aligning findings from different methods.^7^ Software tools, such as MAXQDA’s Segment Matrix, facilitate dynamic display construction, though the analytical thinking required cannot be automated.^4^ Researchers must resist forcing artificial alignment, instead using displays to honestly represent complexity, including divergence and uncertainty.^6^ Documenting this iterative development as part of the integration audit trail captures how understanding evolves through display creation, providing valuable methodological transparency.^14^

Data transformation enables comparison across methodological boundaries through systematic conversion processes.^7^ *Quantitising* transforms qualitative data into numerical form through approaches extending beyond simple frequency counts to include proximity scaling of conceptual distances between themes and intensity rating of expressed sentiments.^9^ Ideally, the transformed results are then integrated with untransformed thematic results. Recent African studies have successfully *quantised* implementation barriers by scoring perceived severity and frequency, enabling statistical comparison across facilities while preserving qualitative insights about barrier characteristics.^5^ Conversely, *qualitising* converts quantitative data into narrative form through case profiling and pattern *narrativisation*.^15^ Statistical clusters become participant typologies with rich descriptive profiles, while numerical trajectories transform into story arcs capturing change over time. These transformation techniques work best when planned during research design rather than imposed during analysis, allowing data collection strategies that facilitate later transformation.^8^

Team-based integration approaches compensate for individual methodological limitations while generating collaborative insights unavailable to solo researchers.^1^ Joint analysis sessions where team members with different expertise examine data together create productive dialogue between methodological perspectives. African research teams increasingly employ structured team integration processes: initial independent analysis preserving methodological integrity, followed by collaborative sessions identifying convergence and divergence, culminating in collective synthesis generating meta-inferences.^3^ Regular team meetings throughout data collection and analysis maintain integration momentum, preventing the common problem of leaving integration until the write-up phases.^14^ Community involvement in team integration sessions ensures findings resonate with local realities while maintaining scientific rigour, particularly important when reconciling biomedical evidence with indigenous health concepts. Practical approaches include engaging community members throughout the research cycle, from initial problem identification through data interpretation and action planning, ensuring shared decision-making and equal power in the integration process. Ethiopian researchers implementing maternal health quality improvement demonstrated this approach through woreda-level joint review forums where community members participated alongside health workers in evaluating quantitative performance data and qualitative implementation experiences, ensuring integrated findings reflected local realities.^17^

Technology considerations significantly impact integration possibilities, though sophisticated software is not a prerequisite for meaningful synthesis.^10^ While Dedoose, MAXQDA, NVivo, QDAMiner and ATLAS.ti offer dedicated integration features, each has strengths and limitations requiring careful alignment with planned strategies.^7^ Practical integration approaches include manual integration using wall displays where teams physically arrange and rearrange printed findings, participatory workshops where interested parties contribute to visual integration, and spreadsheet matrices tracking integration decisions.^4^ The choice between software-based and manual approaches matters less than systematic documentation of integration processes and clear rationales for analytical decisions. Integration success depends more on analytical rigour and creative thinking than on software sophistication.^1^

## A working framework for mixed methods researchers

**FIGURE 1:**
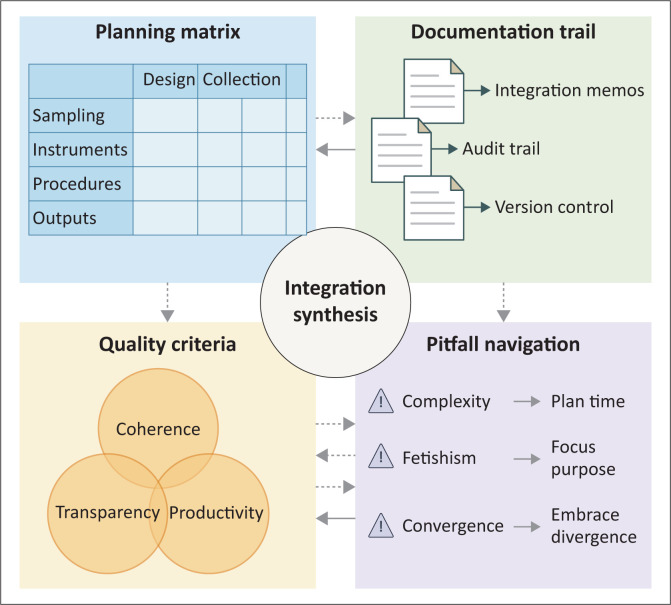
Integration framework for mixed methods researchers.

Mixed methods researchers require systematic frameworks (Figure 1) that transform integration from aspiration to achievement. The step-by-step planning template presented here synthesises successful approaches from African primary care studies with recent methodological advances, providing structured guidance while maintaining flexibility for context-specific adaptation.^6^ Planning begins with interrogating the research problem through integration lens: what aspects require quantitative evidence, what dimensions demand qualitative exploration and most critically, what understanding emerges only through synthesis? These foundational questions generate integration-dependent research questions that cannot be fully answered by either method alone.^1^

The integration planning matrix serves as the central organising tool throughout the research process, mapping integration points across temporal phases and methodological levels.^4^ This matrix captures integration type, procedures, expected outputs and quality criteria for each intersection of research phase with methodological component. Creating the matrix early forces concrete thinking about how methods will connect rather than vague intentions to ‘bring findings together’.^14^ The matrix evolves from planning tool to documentation framework as initial entries become spaces for recording actual integration activities, challenges encountered and adaptations made. Regular updates create a living document capturing the organic development of integrated understanding, transforming potential problems into methodological insights that strengthen research contributions.

Documentation strategies extend beyond the matrix to encompass comprehensive integration audit trails, capturing not just what decisions were made but how and why.^7^ Integration memos provide narrative space for reflexive thinking about challenges and insights, exploring epistemological tensions, documenting breakthrough moments and working through contradictions between findings. These memos prove particularly valuable for developing mixed methods expertise and voice, creating rich material for methodology sections that transcend mechanical description.^10^ Version control systems prevent confusion from accumulating iterations of joint displays and transformed datasets, while cloud-based platforms facilitate team integration while maintaining data security.

Quality assessment requires criteria specifically addressing integration rather than separately evaluating quantitative validity and qualitative trustworthiness. The legitimation framework provides comprehensive criteria, though researchers need practical approaches for ongoing self-assessment rather than post-hoc evaluation.^18^ Integration coherence examines whether strategies align with research questions and whether achieved integration matches intentions. Integration transparency requires clear documentation enabling readers to understand exactly how synthesis occurred, including rationales for choices and honest discussion of challenges.^14^ Integration productivity assesses whether synthesis generated insights beyond single-method contributions, the ‘1+1=3’ criterion distinguishing genuine integration from parallel presentation.^1^

## Common pitfalls and solutions

Integration timing errors derail many mixed methods studies when researchers treat synthesis as an afterthought rather than a continuous process. Studies frequently complete quantitative and qualitative components in isolation, presenting them as independent rather than integrated,^19^ with genuine integration remaining limited even in contemporary research.^20^ This post-hoc approach yields superficial comparisons rather than synergistic insights. The solution requires building integration checkpoints throughout research implementation, with scheduled team meetings where emerging findings inform ongoing data collection. Iterative synthesis during fieldwork allows methodological streams to genuinely inform each other, as demonstrated in recent Ugandan health systems research, where working iteratively between qualitative interviews and observations while focusing on convergence and divergence extended and refined understanding of implementation processes.^21^

Methodological dominance undermines integration when one approach overshadows another, typically with qualitative findings relegated to illustrative vignettes supporting quantitative results.^22^ This hierarchy violates the fundamental mixed methods principle of paradigmatic equality. African primary care research contexts, where biomedical perspectives often dominate, particularly struggle with this imbalance. Solutions involve establishing explicit protocols for equitable treatment of findings, rotating leadership of integration sessions between methodological experts, and using structured integration frameworks that inherently value both approaches equally. Creating joint displays where qualitative themes structure quantitative presentation reverses typical dominance patterns.

Superficial mixing undermines the transformative potential of mixed methods research when integration becomes an afterthought.^19^ Token integration through concluding paragraphs comparing results fails to realise mixed methods potential. Effective solutions include using integrative analytical frameworks from project inception, developing meta-inferences that explicitly bridge methodological contributions, and employing visual integration tools as analytical rather than merely presentational devices.

To illustrate how these strategies work in practice while avoiding common pitfalls, we present a detailed case example from Southern African primary care.

## Case example from African primary care

The InterCARE study in Botswana exemplifies systematic mixed methods integration for adapting an integrated HIV and hypertension care model.^23^ This convergent mixed methods study demonstrates how theory-guided integration generates actionable insights for intervention adaptation in resource-constrained settings. While InterCARE was conducted in HIV clinics integrating hypertension management rather than comprehensive primary care, the integration approaches demonstrated remain transferable across primary care contexts, addressing chronic disease comorbidities.

Integration began with the Consolidated Framework for Implementation Research (CFIR), structuring both data collection and analysis across two clinics. The team collected facility assessments, 100 surveys (patients, treatment partners, healthcare providers, community leaders), and 10 key informant interviews. This framework-guided approach ensured methodological alignment while allowing each method to contribute complementary insights about intervention acceptability and necessary adaptations.

The sampling strategy demonstrated purposeful integration. Survey participants were consecutively recruited for representativeness, while interview participants were purposively selected from survey respondents to maximise perspective diversity. This nested approach enabled quantifying determinants broadly while exploring specific barriers in depth.

Analysis employed systematic convergent comparison organised by CFIR domains. Quantitative data revealed that while 90.3% of patients were taking antihypertensive medications, only 47.2% achieved blood pressure control. Facility assessments identified critical gaps, including non-functioning equipment and inconsistent electricity. These patterns gained meaning through qualitative insights: interviews revealed how ‘a regular cut of electricity [meant] checking of blood pressure may be compromised’, while treatment partners explained how medication stockouts forced patients to travel between multiple clinics.

The integration process identified critical convergence and divergence between data sources. Most interested parties agreed that InterCARE would successfully improve blood pressure control. However, divergence emerged regarding complexity: while 35% of providers surveyed thought adaptation would be difficult, interviews suggested services were already in place and integration would simply bring them together. This nuanced understanding emerged only through systematic comparison across methods.

Integration directly informed intervention adaptations. When both methods revealed insufficient staffing, the team added task-sharing strategies, splitting hypertension management between nurses and doctors. When treatment partners expressed needing additional support, expanded training materials were developed. The CFIR framework enabled mapping specific determinants to targeted implementation strategies. This systematic approach demonstrates that meaningful integration requires not just technical competence but deliberate planning throughout the research process to transform separate data streams into unified understanding.

InterCARE exemplifies the synergistic relationship between Implementation Science and mixed methods research. Implementation Science requires mixed methods to understand both whether interventions work (effectiveness) and how they work in real-world settings (implementation processes).^24^ The CFIR-guided integration represents best practice where quantitative outcomes and qualitative process data must be systematically integrated to generate actionable insights for intervention adaptation.^23^

## Conclusion

This framework demonstrates that meaningful mixed methods integration requires deliberate planning, methodological rigour and intellectual courage to synthesise diverse evidence. Moving from separate streams to confluence demands more than technical competence; it requires embracing complexity, valuing different ways of knowing and developing meta-inferences that transcend disciplinary boundaries. Health researchers must reject superficial mixing that merely satisfies journal requirements, instead pursuing transformative synthesis that generates actionable insights for strengthening health systems.

Future mixed methods research should prioritise capacity building in integration techniques, develop contextually appropriate frameworks that incorporate indigenous knowledge systems, and establish regional networks for sharing integration expertise. The ultimate test remains whether integration improves health outcomes for African communities through deeper understanding of complex health phenomena.

## References

[1] Fetters MD, Freshwater D. The 1+1=3 integration challenge. J Mixed Methods Res. 2015;9(2):115–117. 10.1177/1558689815581222

[2] Harrison RL, Reilly TM, Creswell JW. Methodological rigor in mixed methods: An application in management studies. J Mixed Methods Res. 2020;14(4):473–495. 10.1177/1558689819900585

[3] De Allegri M, Sieleunou I, Abiiro GA, Ridde V. How far is mixed methods research in the field of health policy and systems in Africa? A scoping review. Health Policy Plan. 2018;33(3):445–455. 10.1093/heapol/czx182 29365123 PMC5886233

[4] Guetterman TC, Fetters MD, Creswell JW. Integrating quantitative and qualitative results in health science mixed methods research through joint displays. Ann Fam Med. 2015;13(6):554. 10.1370/afm.1865 26553895 PMC4639381

[5] Bekele A, Alem A, Seward N, et al. Barriers and enablers to improving integrated primary healthcare for non-communicable diseases and mental health conditions in Ethiopia: A mixed methods study. BMC Prim Care. 2024;25(1):211. 10.1186/s12875-024-02458-6 38862874 PMC11167879

[6] Fàbregues S, Younas A, Escalante-Barrios EL, Molina-Azorin JF, Vázquez-Miraz P. Toward a framework for appraising the quality of integration in mixed methods research. J Mixed Methods Res. 2024;18(3):270–280. 10.1177/15586898241257555

[7] Bazeley P. Conceptualizing integration in mixed methods research. J Mixed Methods Res. 2024;18(3):225–234. 10.1177/15586898241253636

[8] Guetterman TC, Manojlovich M. Grand rounds in methodology: Designing for integration in mixed methods research. BMJ Qual Saf. 2024;33(7):470–478. 10.1136/bmjqs-2023-016112 38575310

[9] Zhou Y, Wu M-L. Reported methodological challenges in empirical mixed methods articles: A review on JMMR and IJMRA. J Mixed Methods Res. 2022;16(1):47–63. 10.1177/1558689820980212

[10] Howell Smith MC, Bazis PS. Conducting mixed methods research systematic methodological reviews: A review of practice and recommendations. J Mixed Methods Res. 2021;15(4):546–566. 10.1177/1558689820967626

[11] Kane S, Kok M, Ormel H, et al. Limits and opportunities to community health worker empowerment: A multi-country comparative study. Soc Sci Med. 2016;164:27–34. 10.1016/j.socscimed.2016.07.019 27459022

[12] Fetters MD, Guetterman TC. Development of a joint display as a mixed analysis. In: Onwuegbuzie AJ, Johnson RB, editors. The Routledge reviewer’s guide to mixed methods analysis. New York (NY): Routledge; 2021. p. 259–76.

[13] Fetters MD. The mixed methods research workbook: Activities for designing, implementing, and publishing projects. Thousand Oaks, CA: Sage Publications; 2019.

[14] O’Cathain A, Murphy E, Nicholl J. The quality of mixed methods studies in health services research. J Health Serv Res Policy. 2008;13(2):92–98. 10.1258/jhsrp.2007.007074 18416914

[15] Moran-Ellis J, Alexander VD, Cronin A, et al. Triangulation and integration: Processes, claims and implications. Qual Res. 2006;6(1):45–59. 10.1177/1468794106058870

[16] Ivankova NV. Applying mixed methods in community-based participatory action research: A framework for engaging stakeholders with research as a means for promoting patient-centredness. J Res Nurs. 2017;22(4):282–294. 10.1177/1744987117699655

[17] Tiruneh GT, Zemichael NF, Betemariam WA, Karim AM. Effectiveness of participatory community solutions strategy on improving household and provider health care behaviors and practices: A mixed-method evaluation. PLoS One. 2020;15(2):e0228137. 10.1371/journal.pone.0228137 32023275 PMC7001957

[18] Onwuegbuzie AJ, Johnson RB. The validity issue in mixed research. Res Schools. 2006;13(1):48–63.

[19] Bryman A. Barriers to integrating quantitative and qualitative research. J Methods Res. 2007;1(1):8–22. 10.1177/2345678906290531

[20] Plano Clark VL. Meaningful integration within mixed methods studies: Identifying why, what, when, and how. Contemp Educ Psychol. 2019;57:106–111. 10.1016/j.cedpsych.2019.01.007

[21] Van Hout MC, Zalwango F, Akugizibwe M, et al. Implementing integrated care clinics for HIV-infection, diabetes and hypertension in Uganda (INTE-AFRICA): Process evaluation of a cluster randomised controlled trial. BMC Health Serv Res. 2023;23(1):570. 10.1186/s12913-023-09534-0 37268916 PMC10237070

[22] Hesse-Biber S. Qualitative approaches to mixed methods practice. Qual Inq. 2010;16(6):455–468. 10.1177/1077800410364611

[23] Gala P, Ponatshego P, Bogart LM, et al. A mixed methods approach identifying facilitators and barriers to guide adaptations to InterCARE strategies: An integrated HIV and hypertension care model in Botswana. Implement Sci Commun. 2024;5(1):67. 10.1186/s43058-024-00603-x 38902846 PMC11188218

[24] Proctor E, Silmere H, Raghavan R, et al. Outcomes for implementation research: Conceptual distinctions, measurement challenges, and research agenda. Adm Policy Ment Health. 2011;38(2):65–76. 10.1007/s10488-010-0319-7 20957426 PMC3068522

